# Pressure-selective modulation of NMDA receptor subtypes may reflect 3D structural differences

**DOI:** 10.3389/fncel.2012.00037

**Published:** 2012-09-11

**Authors:** Amir Mor, Yosef Y. Kuttner, Shiri Levy, Merav Mor, Michael Hollmann, Yoram Grossman

**Affiliations:** ^1^Faculty of Health Sciences and Zlotowski Center for Neuroscience, Department of Physiology, Ben-Gurion University of the NegevBeer-Sheva, Israel; ^2^Bioinformatics Core Facility, National Institute for Biotechnology in the Negev, Ben-Gurion University of the NegevBeer-Sheva, Israel; ^3^Faculty of Chemistry and Biochemistry, Department of Biochemistry I – Receptor Biochemistry, Ruhr University BochumBochum, Germany

**Keywords:** 3D model, ion channel modulation, HPNS, magnesium, NMDA receptor

## Abstract

Professional deep-water divers exposed to high pressure (HP) above 1.1 MPa suffer from High Pressure Neurological Syndrome (HPNS), which is associated with CNS hyperexcitability. We have previously reported that HP augments *N*-methyl-D-aspartate receptor (NMDAR) synaptic responses, increases neuronal excitability, and potentially causes irreversible neuronal damage. We now report that HP (10.1 MPa) differentially affects eight specific NMDAR subtypes. GluN1(1a or 1b) was co-expressed with one of the four GluN2(A–D) subunits in *Xenopus laevis* oocytes. HP increased ionic currents (measured by two electrode voltage clamps) of one subtype, reduced the current in four others, and did not affect the current in the remaining three. 3D theoretical modeling was aimed at revealing specific receptor domains involved with HP selectivity. In light of the information on the CNS spatial distribution of the different NMDAR subtypes, we conclude that the NMDAR's diverse responses to HP may lead to selective HP effects on different brain regions. These discoveries call for further and more specific investigation of deleterious HP effects and suggest the need for a re-evaluation of deep-diving safety guidelines.

## Introduction

Professional divers may suffer from the effects of direct high pressure (HP). Animals and humans exposed to ambient pressure above 1.1 MPa (100 m depth) develop High Pressure Neurological Syndrome (HPNS; Bennett and Rostain, 2003; Grossman et al., 2010), which is characterized by reversible but significant cognitive and motor decrements. At greater depths, myoclonia, convulsions, and seizures occur, indicating CNS hyperexcitability. However, prolonged exposure to HP (through repeated deep dives) may result (although this is still disputed) in permanent memory and motor impairment (Sundal et al., 2006; Troland et al., 2006a,b).

The glutamate *N*-methyl-D-aspartate receptor (NMDAR) has been implicated with CNS hyperexcitability as part of HPNS (Fagni et al., 1987; Daniels and Grossman, 2010). We have recently demonstrated in rat hippocampal brain slices that, under HP conditions, NMDAR synaptic responses are significantly augmented and less susceptible to Mg^2+^ blockade. These effects may lead to hyperexcitability and potentially to neurotoxicity (Mor and Grossman, 2006, 2007, 2010). It is important to note that rats seem to be more resistant than humans to HP. Tremor and convulsion thresholds for rats are at 6 MPa and at 9 MPa, respectively (Brauer et al., 1974). Thus, our past and present experiments have been performed at similar levels of HP; humans are affected at lower pressures (see above). 10.1 MPa is considered a “saturating pressure” for experiments on rat preparations.

The first attempt to directly measure NMDAR currents at HP was made by Daniels et al. (1998). By extracting rat cerebellum non-specific NMDAR mRNA and using a *Xenopus laevis* oocyte expression system, they showed that HP increased the receptors' currents. We recently expanded this research by measuring the currents of discrete NMDAR subtypes similarly expressed in oocytes. Surprisingly, preliminary results of two subtypes showed a selective HP effect (Mor et al., 2008). These data revealed a more complex NMDAR behavior at HP that necessitated further research.

Conventional NMDAR is assembled from different combinations of GluN1 and GluN2 subunits in a tetrameric “dimer of dimers” structure (Furukawa et al., 2005; Paoletti, 2011). GluN3 (A and B) subunits may co-assemble with the former subunits to form triheteromeric GluN1/GluN2/GluN3 or diheteromeric GluN1/GluN3 unconventional NMDARs. To date, there are limited data on the spatial distribution and function of the GluN3 subunits (Paoletti, 2011). Therefore, we concentrated on examining conventional NMDARs that do not contain GluN3 subunits. The GluN1 subunit has eight alternative splicing isoforms: GluN1-1a, GluN1-1b; GluN1-2a, GluN1-2b; GluN1-3a, GluN1-3b; GluN1-4a, GluN1-4b (Collingridge et al., 2009). All “b” isoforms have an extra 21–amino-acid loop (see below). The four GluN2(A–D) subunits are encoded by four different genes [GRIN2(A–D); Paoletti, 2011]. Considerable effort has been invested in understanding NMDAR structure-function relations. One approach is to resolve the NMDAR crystal structure. To date, the full NMDAR structure has not been identified. However, partial structural data are available on the GluN1 and GluN2A subunits' ligand-binding domain (LBD; Furukawa et al., 2005) and on the GluN2B N-terminus domain (NTD; Karakas et al., 2009). Interestingly, Traynelis et al. (1995, 1998 reviewed 2010) have shown that the specific region of the GluN1 NTD (exon 5 insert loop in the −1b variant) reduces the inhibition exerted by Zn^2+^, H^+^ and polyamines on the receptor current. Furthermore, the GluN1 and GluN2 NTDs, which are not part of the LBD, are significant modulators of the ion permeation pathway, most probably through their conformational changes (Karakas et al., 2009), which affect the transmembrane domains (TMD) and possibly the intracellular C-terminus domain (CTD).

To date, there are abundant but incomplete data on the spatial distribution and function(s) of NMDAR subtypes in the mammalian brain (Monyer et al., 1994; Paoletti, 2011). Furthermore, the subunit composition of NMDARs changes during development (Cull-Candy et al., 2001) and differs among various types of neurons (Monyer et al., 1994). Studies on recombinant receptors have revealed how the subunit composition endows each NMDAR subtype with unique biophysical and pharmacological properties. Altogether, those studies have revealed the large diversity in the function of NMDAR subtypes in different regions of the mammalian brain. Therefore, an understanding of the HP modulation of specific NMDAR subtypes will reveal important information on their function in different brain areas and perhaps even in specific neuron types.

The goal of the present study was to directly examine the currents of eight NMDAR subtypes in the absence of CNS network influence. The NTD theoretical 3D structures of selected subtypes were modeled in order to reveal the possible biophysical basis for the selective NMDAR response to HP.

## Materials and methods

### Oocyte preparation

Animal experiments were carried out in accordance with the guidelines laid down by Ben-Gurion University of the Negev's ethics committee for the care and use of animals for experimental work. Naive *Xenopus laevis* oocytes were prepared and maintained in NDE96 solution (at 18°C) containing (in mM): 96 NaCl, 2 KCl, 1 MgCl_2_, 1 CaCl_2_, 2.5 sodium pyruvate, 5 HEPES (pH 7.5), and 50 μg/ml gentamicin.

The oocytes were injected with cRNA for co-expression of one of the four rat GluN2 subunits (A–D, 5 ng) with either GluN1-1a or GluN1-1b (5 ng) subunits. All cRNAs were produced by Prof. M. Hollmann's laboratory (Ruhr University, Bochum, Germany). The NMDAR cDNA accession numbers are: GluN1-1a, U08261; GluN1-1b, U08263; GluN2A, AF001423; GluN2B, U11419; GluN2C, U08259; GluN2D, U08260.

A total of eight different NMDAR subtypes (combinations) were successfully expressed on the oocytes' membrane. After incubation for 3–5 days, individual oocytes were placed in a custom-designed recording chamber and perfused (7–8 ml/min) with a frog physiological solution containing (in mM): 115 NaCl, 2.5 KCl, 1.8 CaCl_2_, 10 HEPES, and zero added Mg^2+^. The rationale for [Ca^2+^]_o_-containing recording solutions is provided in the “Data and Statistical Analyses” section. The solutions were introduced into the pressure chamber by means of a high-pressure pump (“minipump,” LDC Analytical Inc., Riviera Beach, FL, USA).

### Pressure, compression, and decompression

The pressure chamber, perfusion system, helium compression, and the experimental setup are described in detail in Mor and Grossman (2006). Briefly, the experiments were carried out in a pressure chamber (Canty Assoc., NY, USA). HP was attained with compressed helium, a gas that is chemically inert under the experimental pressures (0.1–10.1 MPa). Some controls were taken after pressurization to only 0.2–0.3 MPa since, in many experiments, we lost the control recordings after sealing the chamber or with the first attempt to pressurize. This protocol was used in order to increase our yield, assuming that such low pressure effect (if any) is negligible compared to the 10.1 MPa testing pressure. Rates of compression/decompression varied between 0.5 and 1.0 MPa/min. To avoid transient effects of pressure, recordings were taken under strict temperature conditions (25 ± 1°C) and after at least 15 min of stable recording. This timeframe excludes the time needed for the stabilization of temperature transients of ±4°C during compression and decompression. Decompression was attempted to prove the reversibility of HP effects. At this stage, only one pressure step (from control to 10.1 MPa) was used, to minimize waiting time for equilibration and rundown of the preparation. This pressure step is used routinely in our laboratory to faithfully demonstrate HP effects.

### NMDAR current recordings

**Figure 1:**
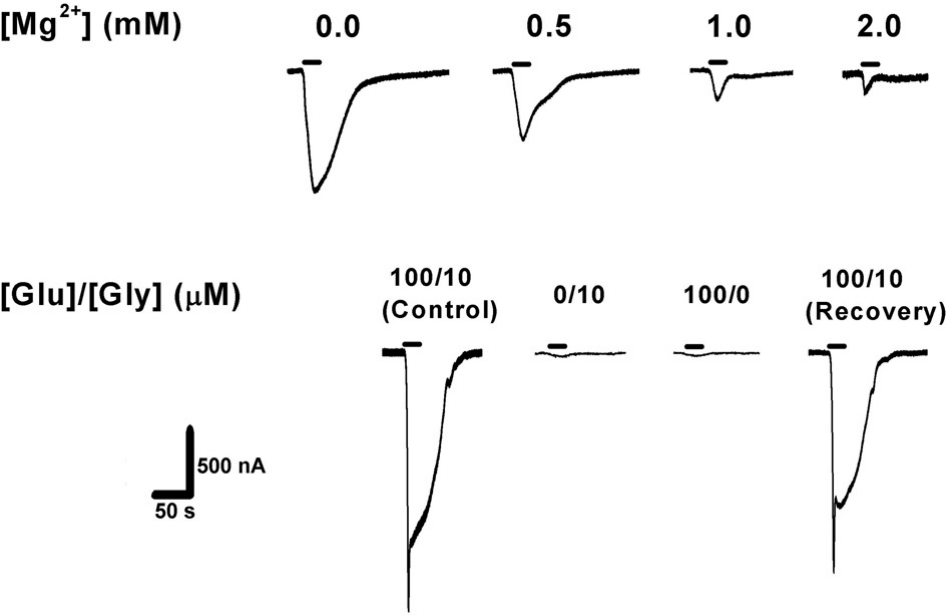
**Confirmation of NMDAR expression in *Xenopus laevis* oocytes. Top:** increasing extracellular [Mg^2+^] blocked NMDAR ionic current in a concentration-dependent manner (expected for all wild-type NMDARs). **Bottom:** activation of NMDAR requires simultaneous application of the co-agonists glutamate (100 μM) and glycine (10 μM), with no [Mg^2+^]_o_ added. Agonist application time was 20 s (horizontal bars). GluN1-1b + GluN2B currents are shown as an example. The same measurements were performed with the other subunit combinations, confirming NMDAR currents in each case.

Oocytes were voltage-clamped at −70 mV employing the two-electrode voltage clamp technique using an Axoclamp-2B amplifier (Molecular Devices, Axon Instruments Inc., CA, USA). The co-agonists glutamate (100 μM, Sigma, Israel) and glycine (10 μM, Sigma, Israel) were added to the physiological solution and applied during 20 s exposure. In order to eliminate the possibility that endogenous functional NMDAR or NMDAR-like proteins would interfere with the recordings, naive oocytes were voltage-clamped and washed with the two agonists. No ionic currents were observed under these conditions (data not shown). It is worth noting, though, that Schmidt and Hollmann (2008) have recently shown that *Xenopus* oocytes can express endogenous *Xen*GluN2 subunits at the protein level, the highest being *Xen*GluN2B. The *Xen*GluN2B subunit by itself will not generate any currents (as above), but, upon expression of heterologous GluN1 subunits in these oocytes, currents may become observable. However, the amplitude of these currents is in the range of only 5–15 nA, which would contribute only a very small percentage of our total observed currents (see Figures 4A,B). Additionally, we confirmed that the recorded currents were NMDAR-mediated as evidenced by the necessity of co-activation by glutamate and glycine, and by the inhibition of the currents by increasing [Mg^2+^]_o_ (Figure 1). Thus, we conclude that “pure” NMDAR currents were recorded (excluding the “fast chloride channels spike”; see below).

As noted above, currents were acquired under control (0.1–0.3 MPa) and hyperbaric (10.1 MPa, compressed helium) conditions, and analyzed offline. Recovery at 0.1 MPa was always attempted. Leak (baseline) currents were subject to change during the compression and decompression procedures and they sometimes differ under hyperbaric versus control conditions. Nonetheless, under constant conditions (pressure, temperature, solution flow rate, pH, etc.), leak currents are stable and thus they could be easily subtracted from NMDAR responses. Oocyte membrane holding potential (−70 mV) was monitored continuously; up to ±1 mV deviations were accepted. Time control protocols for 2–3 h were carried out and showed oocyte stability under control, HP, and decompression conditions (data not shown).

### Experimental data analyses

Classical GluN1 + GluN2 receptors form channels with two conductance levels: a main and a sub-conducting state (for review: Wyneken et al., 2004). Sub-conducting states probably result from fluctuations in the energetics of permeation through a single NMDAR pore. Interestingly, lowering [Ca^2+^]_o_ markedly reduces the frequency of sub-conductance levels (Dravid et al., 2008). This effect can be obtained by replacing [Ca^2+^]_o_ with [Ba^2+^]_o_ (a common procedure used by many researchers). It is already known that such calcium-free solutions enable the acquisition of better NMDAR current recordings with clear steady states due to the reduction of sub-conductance states and the elimination of the fast currents of Ca^2+^-dependent Cl^−^ channels. However, we chose to work with calcium-containing solutions in order to better simulate physiological conditions.

**Figure 2:**
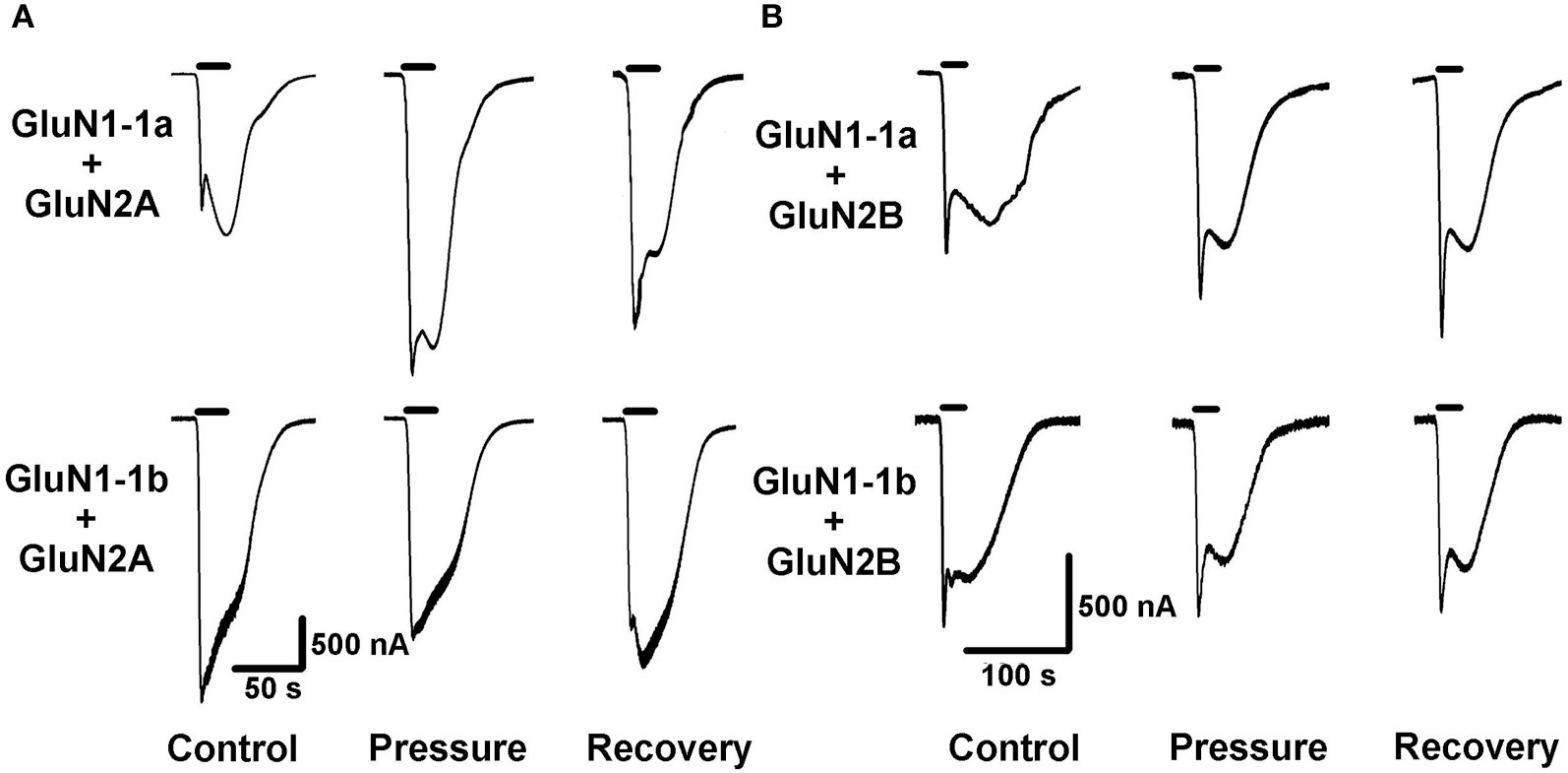
**High pressure (HP) effects on GluN2A and GluN2B NMDAR subtypes. (A)** HP selectively modulates currents of GluN2A subtypes. Top: HP augments GluN1-1a + GluN2A current. Bottom: HP decreases GluN1-1b + GluN2A current. **(B)** GluN2B subtypes GluN1-1a + GluN2B and GluN1-1b + GluN2B are not affected by HP. For all traces: The applied agonist concentrations were 100 μM (glutamate) and 10 μM (glycine) with no [Mg^2+^]_o_ added. The 20 s agonist application time is indicated by horizontal bars. The HP effect is reversed after full decompression for all subtypes.

**Figure 3:**
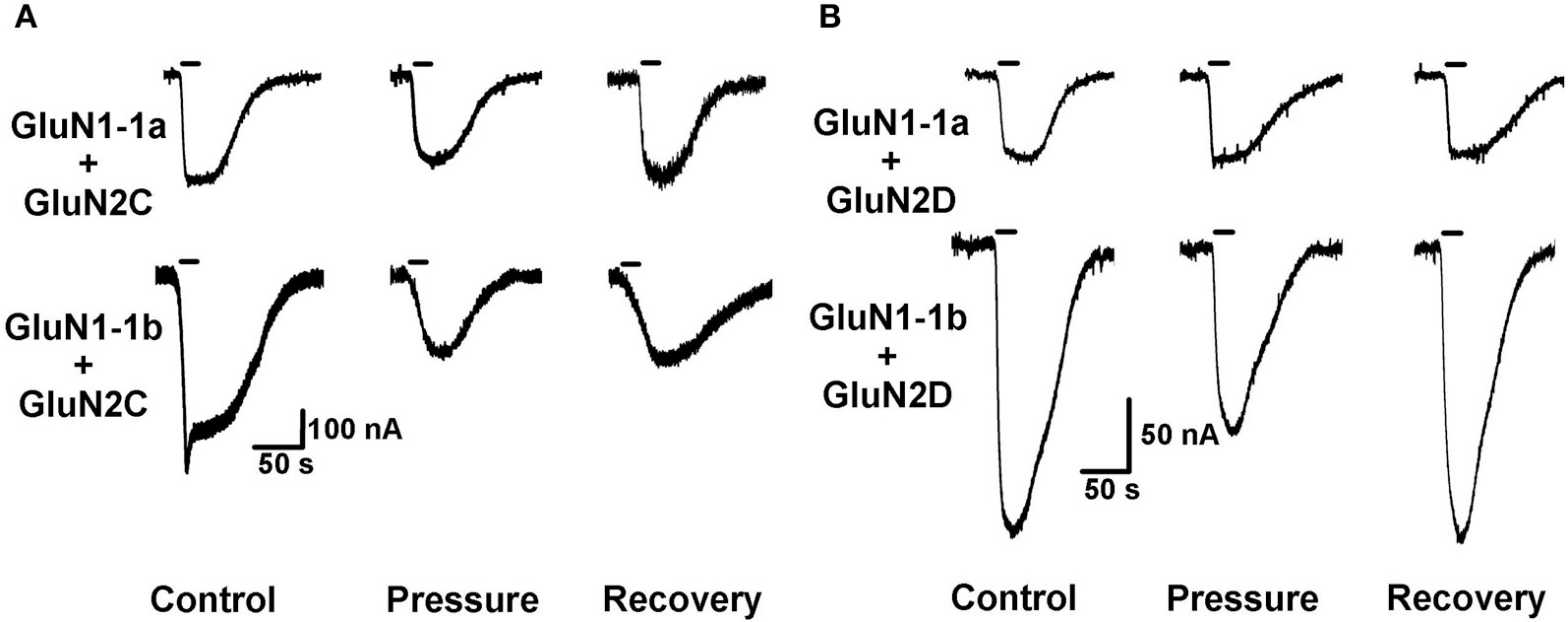
**HP effects on GluN2C and GluN2D NMDAR subtypes. (A)** HP differentially modulates GluN2C subtype currents. Top: HP moderately decreases GluN1-1a + GluN2C current. Bottom: HP greatly decreases GluN1-1b + GluN2C current. Note only partial recovery of the response. **(B)** HP selectively modulates GluN2D subtype currents. Top: GluN1-1a + GluN2D seem to be “pressure-resistant.” Bottom: HP decreases GluN1-1b + GluN2D current. Note complete recovery after a full decompression. For all traces: the applied agonist concentrations were 100 μM (glutamate) and 10 μM (glycine) with no [Mg^2+^]_o_ added. The 20 s agonist application time is indicated by horizontal bars.

The recorded currents were composed of one or two peaks/phases. In the case of two peaks (e.g., Figure 2B), the first relatively fast-appearing and rapidly desensitizing peak probably reflects current flowing through the oocyte's native Ca^2+^-dependent Cl^−^ channels (Leonard and Kelso, 1990). The second “long and delayed peak” represents the NMDAR's steady state, maximal cationic inward current amplitudes. Therefore, only the amplitude of the second peak was measured and analyzed. In the case of a single “long peak” (e.g., Figure 3B), Ca^2+^-dependent Cl^−^ channels were absent or not active (possibly following repeated stimulations). The single peak always appeared after a significant delay. Therefore, it fitted a late NMDAR current component rather than a fast Cl^−^ channel current.

Demonstration of the relative “square” NMDAR currents was difficult to achieve due to two reasons: (1) It was impossible to avoid a relatively large solution volume (“dead space”) inside the HP pump and the tubing. This technical limitation, to a certain extent, reduced our ability to introduce the agonists to the oocytes in an abrupt manner. (2) Ca^2+^ containing solutions were used (see previous paragraph). However, current recordings could be faithfully analyzed because, in each experiment, control and hyperbaric conditions were applied to the same oocyte. In other words, the same oocyte was examined under similar conditions of pH, temperature, solution concentration, flow rate, and agonist concentrations. The only change was the exposure to different pressures. Therefore, confounding factors such as the number of Ca^2+^-dependent Cl^−^ channels, the number/density of expressed NMDARs, and the size of the oocyte could be avoided. Moreover, in each pressure step, the identical agonist application procedure was repeated at least three times, yielding similar and steady current responses.

### Statistical analysis

Due to the fact that each oocyte was used as its own control, and, assuming electrophysiological recordings meet the conditions of a normal distribution, we used paired-sample Student *t*-test analyses. The results of maximal current amplitude measurements are expressed as mean amplitude ± 1 standard error of mean (SEM), with n denoting the number of successful experiments (number of oocytes) for each experimental protocol. The degree of significance is denoted by the values of *p*. Results are considered statistically different when *p* < 0.05. All statistical data were analyzed and calculated using SPSS 13.0 software (SPSS Inc., Chicago, IL, USA). Graphical representations were made by using OriginLab 7.5 software (OriginLab Corp., Northampton, MA, USA).

### Protein structure modeling

#### Sequence alignment

GluN1-1a and GluN1-1b were aligned by Lalign program using a web server running local alignment with default parameters [http://fasta.bioch.virginia.edu/fasta_www2/fasta_www.cgi?rm=lalign] The four GluN2(A–D) were multi-aligned using Clustal W (Larkin et al., 2007).

#### Homology structure modeling

The known bacterial amino acid binding protein, leucine-isoleucine-valine binding protein (LIVBP, PDB coordinates 2LIV; Sack et al., 1989), was used to model the tertiary structure of the GluN2A LIVBP-like NTD following the procedure described by Paoletti et al. (2000). For GluN1 (1a and 1b) subunit homology modeling, the GluN2B NTD crystal structure (PDB coordinates 3JPW; Karakas et al., 2009) was used as a template for modeling. The GluN1 NTD homology models were built using SWISS-MODEL—a fully automated protein structure homology-modeling server (Kiefer et al., 2009). These structures were validated using Procheck (Laskowski et al., 2005), which showed that the model was stereo-chemically significant.

#### Docking

In order to visualize, analyze, and understand the interaction of GluN1-1a with GluN2A, and GluN1-1b with GluN2A, we docked the protein pairs using PatchDock (Schneidman-Duhovny et al., 2005). The result with the biologically most relevant structural and functional significance was selected from the 10 best docking results.

## Results

### Current analyses

**Figure 4:**
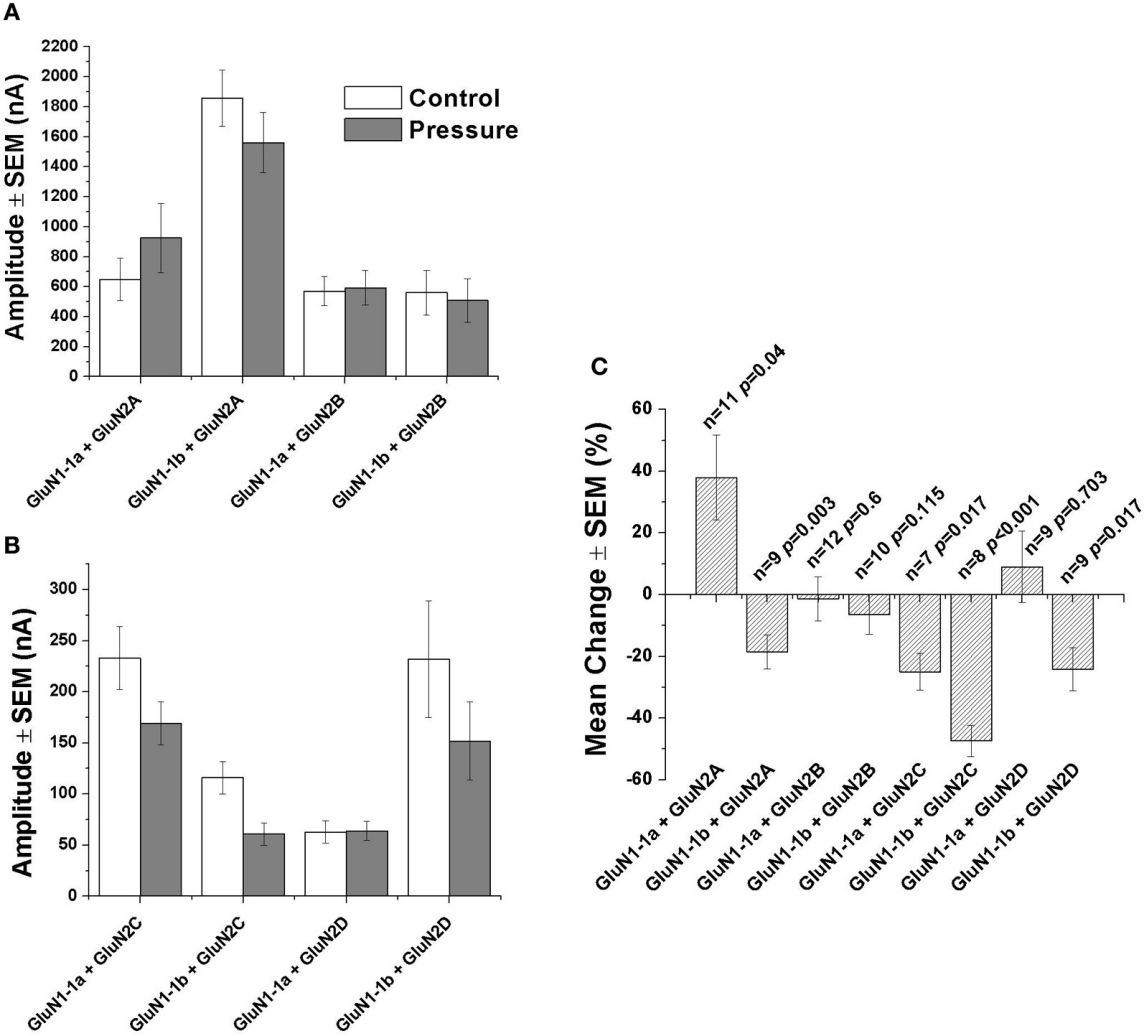
**Statistical analysis of NMDAR currents. (A)** Mean current amplitude under control and hyperbaric conditions (GluN1-1a or GluN1-1b with GluN2A or GluN2B). **(B)** Mean current amplitude under control and hyperbaric conditions (GluN1-1a or GluN1-1b with GluN2C or GluN2D). **(C)** Mean % change of amplitude (calculated for each pair of measurements and averaged). “Control,” 0.1–0.3 MPa; “Pressure,” 10.1 MPa; *n*, number of experiments (oocytes); *p*, degree of statistical significance; SEM, standard error of mean. Statistical tests: paired *t*-test (0.1–0.3 MPa vs. 10.1 MPa).

As expected, GluN2A and GluN2B subtypes containing receptors generated large currents (Figures 2A,B and 4A); GluN1-1b + GluN2A produced the largest currents measured. HP selectively affected the two GluN2A subtypes; HP greatly increased GluN1-1a + GluN2A ionic current, while reducing GluN1-1b + GluN2A currents, thus surprisingly resulting in diametrically opposed response patterns (Figures 2A and 4C). In all GluN2A subtype experiments, a full or near complete recovery was observed.

In contrast with the GluN2A subtypes, the two GluN2B subtypes showed similar behavior; the current amplitude of neither GluN1-1a + GluN2B nor GluN1-1b + GluN2B was significantly affected by HP, or after the decompression process (Figures 2B, 4A and C).

As anticipated, GluN2C and GluN2D subtypes generated relatively small currents (Figures 3A,B and 4B). The currents of the two GluN2C subtypes, GluN1-1a + GluN2C and GluN1-1b + GluN2C, were both depressed by HP, the latter to a greater extent (Figures 3A, 4B and C). However, for unknown reason(s), GluN1-1b + GluN2C subtype currents failed to fully recover after decompression.

GluN2D subtype currents were differentially modulated by HP; GluN1-1a + GluN2D currents were not changed, whereas GluN1-1b + GluN2D currents were significantly depressed by HP (Figures 3B, 4A and C). Following decompression, a full recovery was observed for the GluN1-1b + GluN2D subtype (Figure 3B).

### 3D structure modeling

**Figure 5:**
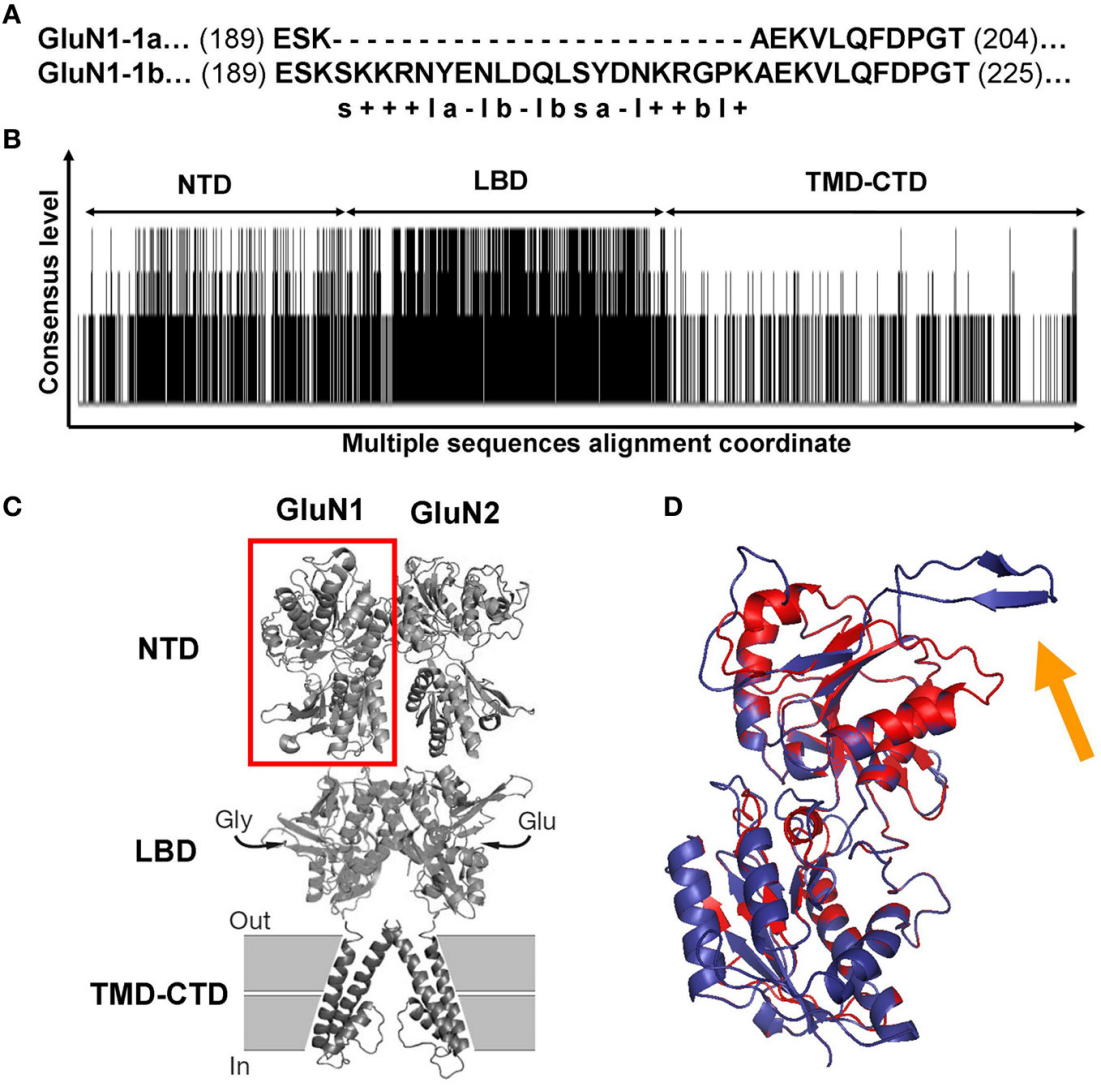
**GluN1 and GluN2 sequence alignments and GluN1 NTD predicted structures. (A)** Sequence alignment of GluN1-1a vs. GluN1-1b. The only difference between the two subunits is the extra 21 amino acids in the GluN1-1b NTD. s, Serine; “+,” positive charge residue; “–,” negative charge residue; l, hydrophilic; b, hydrophobic; a, aromatic. **(B)** Consensus and variation regions of the four GluN2 subunits. Summarized are the results of the multiple sequence alignments of GluN2A, B, C, and D using Clustal W (Larkin et al., 2007). Each black line represents a sequence homology of at least two of the four compared sequences. Line length represents the consensus level. As expected, the LBD is the most conserved. Blank areas represent the absence of sequence homology. **(C)** GluN1-GluN2 dimer model. Red square frame indicates GluN1 NTD. **(D)** Predicted GluN1 NTD tertiary structures. GluN1-1a (red) and GluN1-1b (blue) are superimposed. The orange arrow indicates the loop of the extra 21 amino acids of GluN1-1b. Note that this loop points away from the structure. The figure was created using PyMOL Molecular Graphics System, Version 1.2r3pre, Schrödinger, LLC. NTD, N-terminus domain; LBD, ligand binding domain; TMD-CTD, transmembrane domain and C-terminus domain.

In order to understand the molecular basis for the HP responses of the NMDARs, we first compared the known sequences of the two GluN1-1 subunits. As shown in Figure 5A, GluN1-1a and GluN1-1b have almost identical sequences, yet GluN1-1b contains an additional 21 amino acids (exon 5 insert, starting at position 193) at the NTD (see Figure 5C). These extra amino acids are mainly hydrophilic and many of them are charged (see details in Figure 5A). In contrast, the sequence differences among the GluN2 subunits are large (Figure 5B). There are many areas with a high consensus (e.g., in the LBD), areas with less homology (NTD), and other areas with great diversity (TMD-CTD). These variable sequences may partially underlie the different behavior of various NMDAR subtypes.

**Figure 6:**
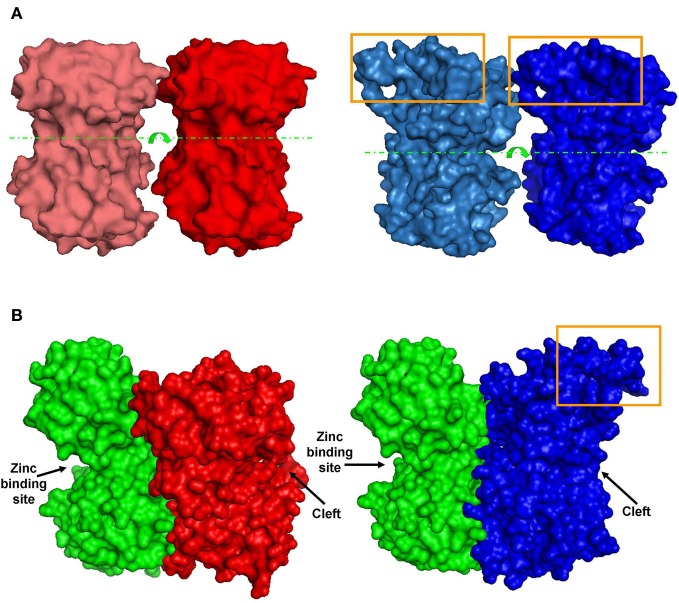
**Predicted dimer structures. (A)** GluN1 homodimers. Left (red)—A 3D model of GluN1-1a↔GluN1-1a NTD interactions. Right (blue)—A 3D model of GluN1-1b↔GluN1-1b NTD interactions (manual docking). Box—the extra 21 amino–acid-loop (exon 5). This structure might interfere with the interaction of GluN1-1b subunits. Broken lines indicate the cleft. Arrows represent the cleft's direction. **(B)** GluN1-GluN2 heterodimer 3D structures. Left—docking of GluN1-1a NTD (red) to GluN2A NTD (green). Right—docking of GluN1-1b NTD (blue) to GluN2A NTD (green). Note that the loop of 21 extra amino acids in GluN1-1b (orange box) faces out and does not interfere with sub-domain interaction. The figure was created using PyMOL.

As a first attempt to understand 3D structure-function relations at HP, we chose to focus only on GluN1-1a + GluN2A vs. GluN1-1b + GluN2A subtypes. This pair is the most interesting because the subtypes were inversely affected by HP despite the fact that they are almost identical; they differ only by one exon insert in the NTD of the GluN1-1b subunit, while the GluN2 subunits are identical. Using homology modeling (see “Materials and Methods”), we built predicted tertiary structures of GluN1-1a and GluN1-1b NTDs. Superimposition of these structures showed that the main difference is a surface loop (depicted by the orange arrow in Figure 5D). Next, following the assumptions of Furukawa et al. (2005) of a “dimer of dimers” structure, we examined the interaction of the subunit variants among themselves. We modeled GluN1-1a↔GluN1-1a and GluN1-1b↔GluN1-1b homodimers (Figure 6A) as well as GluN1-1a↔GluN2A and GluN1-1b↔GluN2A heterodimers (Figure 6B). The extra loop appears to interfere with the interaction of subunits in the GluN1-1b↔GluN1-1b homodimer (Figure 6A). However, in the GluN1-1b↔GluN2A heterodimer 3D model, the loop faces outward and does not interfere with any sub-domain interactions (Figure 6B). At this point, we were unable to calculate a 3D model prediction of the NMDAR NTDs tetramer due to software limitations and the lack of a fully resolved NMDAR crystal structure.

## Discussion

The NMDAR has been repeatedly implicated in HPNS generation. The evidence accumulated to date indicates that at HP the NMDAR response is increased (Fagni et al., 1987; Mor and Grossman, 2006, 2007, 2010). This augmentation is thought to be one of the key elements causing HPNS and possibly long-term irreversible CNS impairment.

### Distribution of subunits: local effects

Our present study together with our preliminary work (Mor et al., 2008) indicate a more complex picture of NMDAR-mediated HP effects. Of the eight NMDAR subtypes examined, only one, GluN1-1a + GluN2A, produces significantly larger ionic currents under HP conditions. Three subtypes are “HP-resistant” and four were significantly depressed by HP. Since NMDAR subunit distribution in the brain may vary, HP-dependent NMDAR hyperexcitability in the CNS (with possible neurotoxicity) is expected to be region-specific. For example, the GluN1-1a and GluN1-1b variants have largely overlapping CNS expression patterns but the “1a” forms outnumber the “1b” forms in most, but not all, brain regions (expression level ratio 5:1, respectively, in the forebrain but 1:5 in the cerebellum). Notably, in the hippocampus, while the GluN1-1a isoform is expressed at high levels in all principal cell layers (in the dendate gyrus and CA1-3 regions), the GluN1-1b isoform is largely restricted to the CA3 layer (Paoletti, 2011). Dominant NMDAR subtypes in the mammalian hippocampal CA1 region are GluN1-1a + GluN2A and GluN1-1a + GluN2B. If HP augments the former and does not affect the latter, the net effect would be response augmentation as indeed shown in our previous studies (Mor and Grossman, 2006, 2007, 2010). This increase may lead to the hippocampal hyperexcitability (see “Introduction”) and the epileptiform activity in the whole animal observed by Fagni et al. (1985). It is worth mentioning that once epileptiform activity has begun, brain regions with a preponderance of “HP-resistant” or “HP-depressed” NMDAR subtypes may no longer be protected. The observed increase of the inward current via the most abundant NMDAR subtype may also offer an explanation for the permanent CNS impairment reported for professional divers. Such impairment may be mediated through an increased Ca^2+^ influx (via NMDARs) into the neurons (Cull-Candy et al., 2001). The repeated deep dives could expose an increasing number of CNS neurons to this deleterious effect; with time, the loss of neurons may reach a critical level of clinical manifestation(s).

### Subunits structure: differential effects

The observed HP selectivity raises the question of what could be the possible physical site(s) that may promote conformational changes favoring NMDAR channel conductance augmentation or attenuation. We scanned the amino acids sequences of selected NMDAR subunits. GluN2(A–D) sequences differ considerably (exclusive of their LBD sequences, Figure 5B). Therefore, at this stage it would be impractical to look for NMDAR functional properties through GluN2 structural differences until additional information regarding the full NMDAR crystal structure becomes available. As an alternative approach, we made an attempt to explain HP selectivity by restricting our comparison to the 3D structures of GluN1-1a + GluN2A and GluN1-1b + GluN2A receptors that differ only in the single 21 amino acid loop of the GluN1-1b exon 5 insert in the NTD, which is absent in GluN1-1a variant. Since these two subtypes are inversely affected by HP, the difference should be attributed, by default, to the extra loop. Therefore, we modeled only the NTDs of the GluN1-1a and GluN1-1b and tried to dock them either as homodimers or heterodimers (see Figure 6) since the exact relations in the receptor tetramer are not clear. The insert loop appears to interfere with subunit interactions in the GluN1-1b↔GluN1-1b homodimer but not in the GluN1-1b↔GluN2A heterodimer. Due to the lack of exon 5 expression, the homodimer GluN1-1a↔GluN1-1a has less impairment in subunit interaction. It is important to note that Traynelis et al. (1995, 1998) have reported that GluN1-1b, −2b, −3b, and −4b splices, which contain the extra loop (see “Materials and Methods”), are very potent in inhibiting the Zn^2+^, H^+^ (pH), and polyamine-induced depression of NMDAR currents. In contrast, GluN1-1a, −2a, −3a, and −4a, which lack this loop, do not have that capability. They even suggested that all three mechanisms may converge on a single site. Furthermore, they have shown that this behavior is restricted to NMDAR combinations with GluN2A and GluN2B but not with GluN2C and GluN2D. These two features, namely, the differential effect of the GluN1-b vs. GluN1-a splice variants and the selectivity for specific GluN2 combinations, are very similar to our present results. We may postulate, therefore, that all the combinations of GluN1-Xb (i.e., GluN1-1b, or GluN1-2b, or GluN1-3b, or GluN1-4b with GluN2A) will be depressed by HP and that all GluN1-Xa (i.e., GluN1-1a, or GluN1-2a, or GluN1-3a, or GluN1-4a with GluN2A) will be potentiated by pressure. This hypothesis will be the subject of future research. Another support for the specific role of GluN1-b variants comes from the observation that the exon 5 loop contributes to the receptor deactivation, resulting in an acceleration of the current's decay time course (Rumbaugh et al., 2000).

Unfortunately, due to incomplete crystal structure data, we were unable to model the complete tetrameric NTD structure. Nevertheless, based on the limited 3D structure model and the information on receptor function, we may postulate the following: The GluN1-1b NTD extra loop interferes with inter-subunit interactions and that might result in reduced inhibition on the channel pore. Consequently, under control conditions, GluN1-1b + GluN2A currents are much larger when compared to GluN1-1a + GluN2A currents (Figures 4A,C). However, under HP conditions, a small local conformational change in the loop may significantly reduce the channel's open state probability (in our experiments about 18% reduction in current). In accordance with that model, three of the four tested GluN1-1b + GluN2 combinations were similarly depressed at HP while only one was not significantly affected.

It is more difficult to suggest an underlying mechanism for the rarely observed increase in receptor conductance under HP conditions. Yet, such an increase would not be a single observation since an increase in ionic currents of voltage-dependent potassium channels (in invertebrate neurons) and of specific L-type (Ca_V_1.2) Ca^2+^ channels (expressed in *Xenopus* oocytes) has been reported (for review see Aviner et al., 2010). We postulate that HP disrupts the relatively stable inter-subunit interactions in the GluN1-1a homodimer due to, for example, changes in local charge movement or water molecule rearrangement. Consequently, the GluN1-1a homodimer may be less stabilized and promote greater channel conductance. In contrast, in a single channel recording from an acetylcholine receptor at high hydrostatic pressure (Heinemann et al., 1987) it was found that the maximal conductance of the channel is pressure-resistant, while the kinetics of the channel opening is significantly slowed. The Ach receptor 3D structure is completely different from that of the NMDARs; therefore an immediate inference could not be made. Since the pressure effect could be differential, it is reasonable to assume that, in some splice variants of the NMDARs, pressure may also reduce the channel's open state probability.

However, at this stage, we are unable to determine whether the surprising observations result from a change in allosteric inhibitory effect (like the -1b loop control), direct alternation of conductance through the channel pore, functional modification of auxiliary protein(s) (Neto1; Ng et al., 2009), or membrane lipid-channel protein interaction. Future studies with site-directed mutagenesis (such as substitution of amino acids involved in the -b loop activity; Traynelis et al., 1995, 1998), single channel recordings, and further NMDAR crystallographic data at HP are necessary in order to fully answer these questions.

In conclusion, even without fully understanding the exact mechanism(s) of pressure effects, our data support the postulated NMDAR involvement in HPNS hyperexcitability and suggest a possible cause for the suspected long-term HP health effects. Moreover, our findings demonstrate a selective role for the specific combination(s) of receptor subunits. Thus, the physiological consequences of pressure exposure are also dependent on the NMDAR subtype distribution in the brain. It has already been reported that subtype-specific allosteric inhibitors of NMDAR exhibit much fewer side-effects than broad-spectrum NMDAR antagonists (direct channel blockers or competitive antagonists; Traynelis et al., 2010). There is growing interest in the therapeutic potential of compounds capable of fine-tuning the activity of specific NMDAR subtypes. It is hoped that, with additional 3D modeling of the various NMDAR subtypes, it will be possible to develop subtype-targeted medications, which will antagonize or at least reduce the negative HP effects on divers' performance and health. These discoveries also call for cautious consideration of safety procedures (e.g., frequency of diving, exposure time, and depth limitations) for repeated deep diving of commercial divers in order to reduce the postulated accumulating deleterious effects of HP.

## Author contributions

Yoram Grossman and Amir Mor conceived the study and designed the electrophysiological experiments. Experiments were carried out and analyzed by Amir Mor. The 3D modeling was created, designed, and analyzed by Yosef Y. Kuttner. Shiri Levy, Merav Mor, and Michael Hollmann contributed to the analysis and interpretation of the data presented in this paper. The manuscript was written by Amir Mor, Yosef Y. Kuttner, and Yoram Grossman and revised with the critical appraisal of Michael Hollmann. All authors approved the final version for publication.

### Conflict of interest statement

The authors declare that the research was conducted in the absence of any commercial or financial relationships that could be construed as a potential conflict of interest.

## Abbreviations

CNS: central nervous system
CTD: C-terminal domain
HP: high pressure
HPNS: high pressure neurological syndrome
LBD: ligand binding domain
NMDA: *N*-methyl-D-aspartate
NMDAR: NMDA receptor
NTD: N-terminal domain
TMD: transmembrane domain.
